# The upshot of Polyphenolic compounds on immunity amid COVID-19 pandemic and other emerging communicable diseases: An appraisal

**DOI:** 10.1007/s13659-020-00271-z

**Published:** 2020-10-15

**Authors:** Ayman Khalil, Diana Tazeddinova

**Affiliations:** Department of Food Technology, South Ural State University, Chelyabinsk, Russian Federation

**Keywords:** Polyphenols, Natural product, COVID-19, SARS, Respiratory tract, Infectious diseases

## Abstract

Polyphenols are a large family of more than 10,000 naturally occurring compounds, which exert countless pharmacological, biological and physiological benefits for human health including several chronic diseases such as cancer, diabetes, cardiovascular, and neurological diseases. Their role in traditional medicine, such as the use of a wide range of remedial herbs (thyme, oregano, rosemary, sage, mint, basil), has been well and long known for treating common respiratory problems and cold infections. This review reports on the most highlighted polyphenolic compounds present in up to date literature and their specific antiviral perceptive properties that might enhance the body immunity facing COVID-19, and other viral infectious diseases. In fact, several studies and clinical trials increasingly proved the role of polyphenols in controlling numerous human pathogens including SARS and MERS, which are quite similar to COVID-19 through the enhancement of host immune response against viral infections by different biological mechanisms. Thus, polyphenols ought to be considered as a potential and valuable source for designing new drugs that could be used effectively in the combat against COVID‐19 and other rigorous diseases.

## Introduction

Since the first unveiling of the novel coronavirus (COVID-19) in the city of Wuhan, Hubei province, China in late December 2019, exceptional and unprecedented dares presented by the rapidly rising of COVID-19 pandemic health crisis are confronting people worldwide. COVID-19, a single-stranded RNA virus is actually complicated by severe acute respiratory syndrome coronavirus 2 (SARS-CoV-2), a newly identified virus alike SARS-CoV and MERS-CoV-2 that cause the severe acute respiratory syndrome and Middle East respiratory syndrome respectively [1]. The main symptoms of COVID-19 comprise fever, dry cough, difficulty in breathing, fatigue, mild dyspnoea, sore throat, headache, conjunctivitis and gastrointestinal problems [2–4]. Initially, the pathogen strikes the respiratory tract particularly, the lower respiratory part, leading to a severe lung injury at all ages, subsequently in elderly people and those immuno-compromised individuals in particular, it may lead to a severe pneumonia associated with a systemic and strong inflammation implicated in airway damages, acute respiratory distress syndrome (ARDS) and subsequently, multi-organ failure and cause fatality [4, 5]. During SARS-CoV-2 infection, the viral immune response is divided into two main phases: incubation (non-severe stages), and massive destruction (severe stages). In the first phase, a specific adaptive immune response is vital to remove the virus and prevents the initiation of the second phase of virus infection from commencing. Therefore, good general health and appropriate genetic background of the host is crucial for the development of an endogenous protective immune response at the incubation and non-severe stages that produces specific antiviral immunity. However, in the case of an impaired immune response (second phase), virus will spread and massive destruction of the affected tissues will occur, followed by an induced innate inflammation in lungs mediated by pro-inflammatory macrophages, granulocytes, generation of pro-inflammatory cytokines and chemokines such as IL-6 and IP-10, macrophage inflammatory protein 1 such as MIP1α, MIP1β and MCP1. These inflammatory molecules attract monocytes, macrophages and T cells to the site of infection, promoting further inflammation creating overproduction of pro-inflammatory cytokines, which ultimately damage the lung structure and then the resulting cytokine storm enters the circulation system and reaches other organs, leading to a multi-organ damage [4, 6, 7]. Actually, data indicated that activation of the nuclear factor (NF)-κB transcription factor (NF-κB) signaling pathway represents a major contribution to the inflammation induced post SARS-CoV infection and that NF-κB inhibitors are promising antiviral drugs against infections caused by the virus and potentially other pathogenic human coronaviruses [8]. In this regards, several clinical trials based mainly on anti-oxidant, anti-inflammatory, immunomodulatory drugs and other therapies are conducted to find a possible COVID-19 treatment [9]. According to world health organization (WHO), appropriate nutrition and well-balanced diet are vital to be healthier with stronger immune systems and lower risk of chronic illnesses and infectious diseases. Since, the human immune response has often been shown to be weakened by insufficient nutrition in several model systems [10]. Therefore, nutrition intervention and therapy need to be considered as an integral part of the approach to patient’s victim of COVID-19 infection especially among the general health workers [3, 11]. In fact, several epidemiological studies have repeatedly shown an inverse association between the risk of chronic human diseases and the consumption of polyphenolic rich diet [12]. In fact, polyphenols, essential micronutrients in human diet, constitute one of the most numerous and ubiquitous group with more than 8000 polyphenolic compounds identified in the plant kingdom. In the past decades, polyphenols (phenolic acids, flavonoids, stilbenes, and lignans [13] have been intensively studied due to their countless benefits for human health particularly, their strong anti-oxidant and anti-inflammatory proprieties [14]. Moreover, the newly emerging diseases such as the novel COVID-19 and other infectious diseases that impose grand burden on both human health and economy worldwide, require urgent research for a novel antiviral drug preferably from a natural origin such as polyphenols [15, 16]. The polyphenols antiviral effect is due to the interactions between the phenyl rings and viral proteins and/or RNA, or via their capacity to interfere with the host cell defense by regulating MAP kinase signaling [17, 18]. It is noteworthy that the antiviral effect of polyphenols is not significantly affected by viral mutations due to their binding capacity to the viral envelope lipids or sugar moieties [19]. These enormous features of polyphenols as a natural economical, simple and environmental friendly compounds warrant the birth of this review that highlights the current state of knowledge regarding the most important polyphenolic compounds [20], their relative importance, their general properties in human health focusing principally on their antiviral activities, and their role in enhancing immunity in order to face the emergence of several infectious diseases such as COVID-19 and its related symptoms.

## Quercetin

**Fig. 1:**
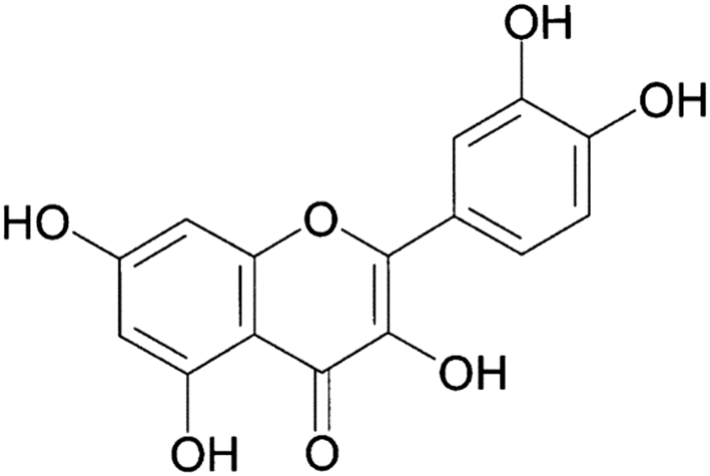
Structure of quercetin (3,3′,4′,5,7-pentahydroxyflavone)

Quercetin (3,3′,4′,5,7-pentahydroxyflavone) (Fig. 1), is a natural plant flavonoid involved in many plant processes such as pigmentation and protection against bacteria and fungi [21].

Quercetin and its glycosylated (quercetin-3-*O*-β-D-glucuronide) forms represent 60–75% of flavonoid intake [22] and it has been found ubiquitously in many fruits and vegetables including apples, berries, brassica vegetables, capers, grapes, red onions, shallots, kale, green tea, cranberries, broccoli, tomatoes, nuts, flowers, barks, leaves and honey [23, 24]. Quercetin has been known for its antiviral, anti-inflammatory, anti-carcinogenic and antioxidant properties [25] as well as its exceptional ability to improve mental and physical activities and decrease infection risk [26]. In fact, quercetin is considered as a strong anti-oxidant due to its ability to inhibit lipid peroxidation and thus it protects many body organs from harmful effects of the consequential free radicals [27, 28]. In addition, the general mechanism by which quercetin exerts its anti-inflammatory effects is believed to be due to its ability to restrain COX-2 and iNOS, NF-κB, activator protein-1 (AP-1), and mitogen-activated protein kinase (MAPK) usually, associated with the inflammatory response during infections. Subsequently, inhibiting the production of pro-inflammatory markers and cytokines (IL-1β, IL-6, IFN-γ, TNF-α, monocyte chemoattractant protein-1: MCP-1, lipoxygenase: LOX) and thus enhancing the anti-inflammatory cytokines like IL10 [29]. Moreover, a study demonstrated that both quercetin and quercitrin could inhibit LPS-induced macrophage inflammation and oxidative stress by experiment and theoretical calculations [30]. Quercetin blocks in vitro TNFα-mediated inflammation which prevents TNF-α from directly activating extracellular signal-related kinase (ERK), c-Jun NH2-terminal kinase (JNK), c-Jun, and NF-κB, which are well known potent inducers of inflammatory gene expression and protein secretion. Quercetin can also indirectly inhibit the inflammatory process by increasing peroxisome proliferator-activated receptor c (PPARγ) which is antagonized NF-kB and AP-1 thus inhibiting the inflammatory cascades and subsequently decreases the expression of the inflammatory genes [31]. Quercetin has also beneficial immuno-stimulatory effects by inducing the expression of several vital genes and the production of Th-1 derived IFN-γ and down-regulating Th-2 derived interleukin 4 (IL-4). In fact, quercetin is seen as a universal suppressor of the accumulation and activation of immune cells, which prevents chronic inflammation [31, 32]. Quercetin relaxes airway smooth muscle and potentiates β-agonist-induced relaxation via dual phosphodiesterase inhibition of purified phosphatidylinositol-specific phospholipase (CPLCβ) and phosphodiesterase type 4 inhibitor (PDE4) [33]. Quercetin has exerted strong anti-pathogenic effects against several causal agents of upper respiratory tract infection in vitro studies [34]. Quercetin inverted lung fibrosing in mice and reversed the disease progression normally caused by usual pulmonary aging markers [34]. Moreover, it was found to reduce the reactive oxygenated species (ROS) produced during viral infection and subsequently decrease pro-inflammatory markers such as IL-8, TNF-α, IL-1β and IL-6 [25] and increases anti-inflammatory cytokines such as IL-10 [35], indicating that it has clear antiviral effects on several respiratory and common cold viruses through its ability to reduce virus imputation, replication and viral load in vitro, as well as lung inflammation and airways hyper-responsiveness in vivo [29]. Quercetin was found to be a strong inhibitor of influenza A virus in the early stage of infection [36] and demonstrated significant inhibitory activities against dengue virus type-2 but the mechanisms of how quercetin exerts its antiviral effects are not fully understood [37]. Quercetin prevented rhinovirus-induced progression of lung disease in mice with chronic obstructive pulmonary disease (COPD) [38]. Quercetin-3-β-galactoside, a quercetin derivate, was found to inhibit in vitro a protease essential for viral replication of SARS-CoV [39] and to block the enzymatic activity of MERS‐CoV 3CLpro [40]. Moreover, quercetin has been reported to inhibit the proteolytic activity of SARS-CoV 3CLpro [41] from the same family as COVID-19. Therefore, these properties of quercetin could render it a favorable natural compound to be exploited in the medicinal field due chiefly to its strong antioxidant properties [42].

## Catechins

**Fig. 2:**
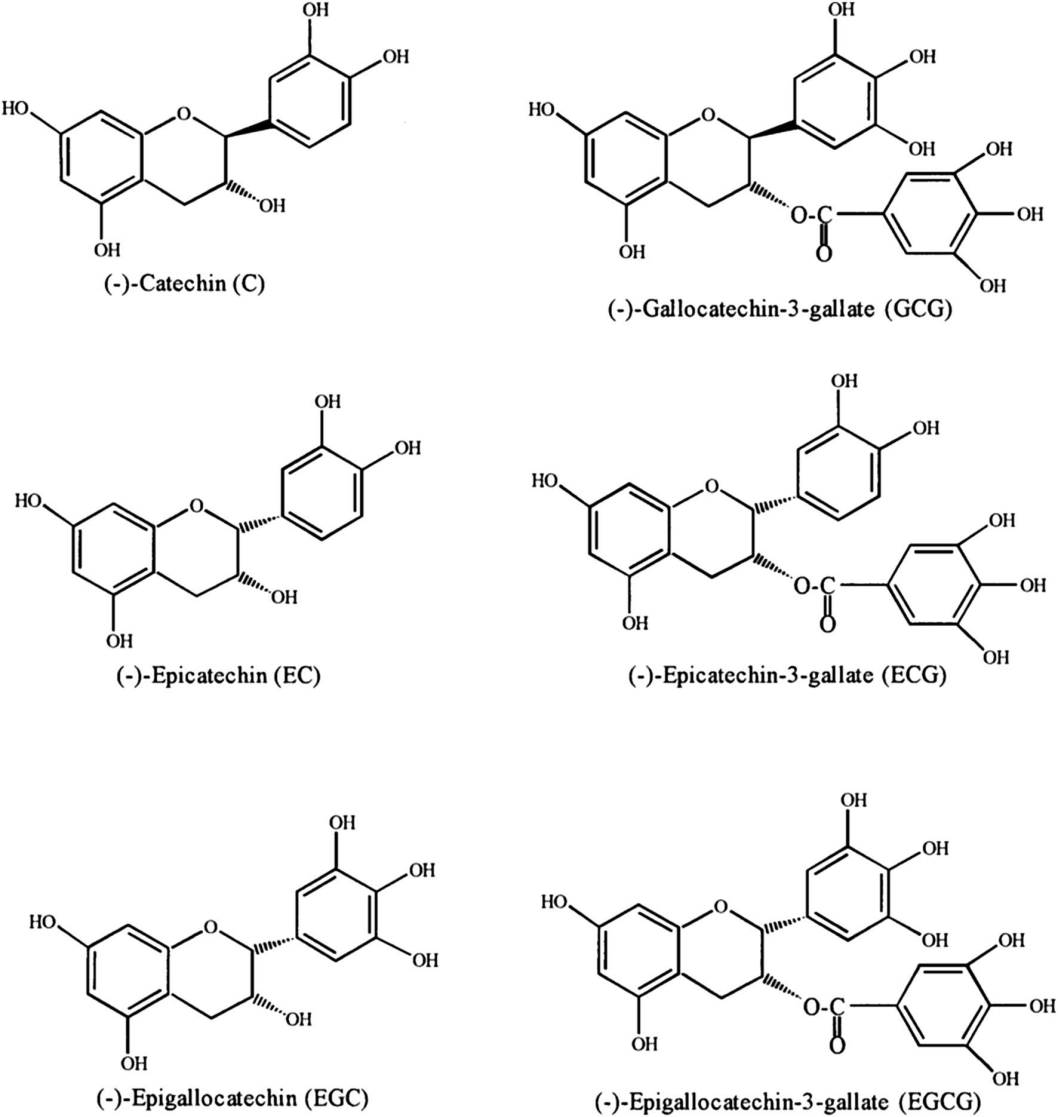
Structure of catechins: catechin [( +)-3,3′,4′,5,7-pentahydroxyflavan)], epicatechin (EC) [(−)-3,3′,4′,5,7-pentahydroxyflavan], epicatechin gallate (ECG) [C_22_H_18_O_10_], epigallocatechin (EGC) [(−)-3,3′,4′,5,5′,7-hexahydroxyflavan], gallocatechin (GC) [C_15_H_14_O_7_], epigallocatechin gallate (EGCG) [C_22_H_18_O_11_] and gallocatechin gallate (GCG) [C_22_H_18_O_11_]

Catechins (Fig. 2) are natural flavonoid polyphenolic compounds that primarily include, catechin [(+)-3,3′,4′,5,7-pentahydroxyflavan)], epicatechin (EC) [(−)-3,3′,4′,5,7-pentahydroxyflavan], epicatechin gallate (ECG) [C_22_H_18_O_10_], epigallocatechin (EGC) [(−)-3,3′,4′,5,5′,7-hexahydroxyflavan], their stereoisomer gallocatechin (GC) [C_15_H_14_O_7_] and epigallocatechin gallate (EGCG) [C_22_H_18_O_11_] [43].

Catechins present in many dietary products, medicinal plants, and fruits including apples, blueberries, gooseberries, grape seeds, kiwi, strawberries, green tea, red wine, beer, cacao liquor, chocolate, cocoa. Catechins particularly, those found in green tea (mainly EGCG and EGC) have many favorable properties for human health and were confirmed in vivo and in vitro as an anticancer, anti-obesity, antidiabetic, anti-inflammatory, anti-cardiovascular, anti-infectious, hepatoprotective, and neuroprotective [44]. Catchins of green tea have been shown to decrease denaturation of tissue proteins, ROS, free radical and NO productions. On the contradictory, they were found to increase the production of anti-inflammatory cytokines and antioxidant enzymes [45, 46]. In fact, catchins exert a strong anti-inflammatory property due to their ability to activate/deactivate inflammation-related oxidative stress-related cell signaling pathways, such as NF-κB, MAPKs, transcription factor nuclear factor (erythroid-derived 2)-like 2 (Nrf2), signal transducer and the activator of transcription 1/3 (STAT1/3). This ability of catchins to interact with those pathways might contribute to their role in inhibiting the infiltration and proliferation of immune-related cells and regulate inflammation and oxidative reactions by lowering of the production of pro-inflammatory cytokines such as IL-17, chemokines, adhesion molecules, and inflammatory-related enzymes, like iNOS and COX-2, augmenting the production of the anti-inflammatory cytokines such as IL-10, and suppress many oxidative stress-related pathways responsible for the inflammation processes [43, 47].

Several studies have indicated the role of catchins in preventing infectious diseases [48]. Regular consumption of green tea has been shown to decrease influenza infection and some cold symptom rates. In addition, gargling with tea catechins protects against influenza infection and common cold [49]. A study has also shown that consuming green tea supplements twice daily for 3 months resulted in a significant decrease in cold or influenza symptoms by approximately 23%. Moreover, green tea consumption per day or per week was in reverse to the instance numbers of influenza A or B among school children in Japan [50]. Catechin compounds were also shown to exhibit high binding affinity to Papain-like proteinase (PLpro) which is involved in RNA SARS-CoV-2 replication, suggesting the potential effectiveness of these compounds in the treatment of SARS-CoV-2 [51, 52]. EGCG derivatives such as EGCG-fatty acid monoesters were found to be as an innovative approach to the prevention of viral infections including emerging fatal viruses, such as Ebola virus, SARS virus and MERS virus [53]. In fact, this action is probably due to their increased affinity for viruses and cellular membranes [54]. Catechins as the major content of green tea may be a potential treatment of COVID-19, caused by the new coronavirus that is nominated as severe acute respiratory syndrome-related coronavirus SARS-CoV-2 especially, the breath shortens and other symptoms related to this emerging virus infection.

## Rutin

**Fig. 3:**
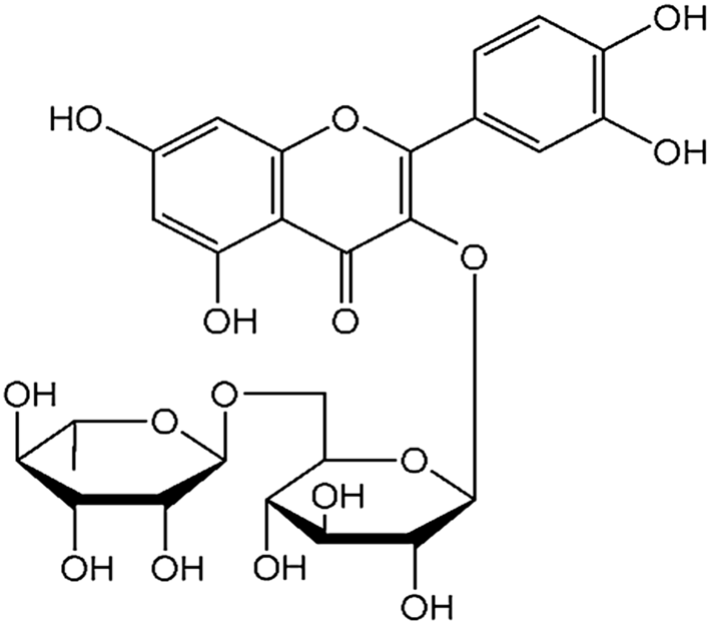
Structure of rutin (3,3′,4′,5,7-pentahydroxyflavone-3-rhamnoglucoside)

Rutin (3,3′,4′,5,7-pentahydroxyflavone-3-rhamnoglucoside) (Fig. 3), is another important naturally occurring flavonoids also known as rutoside, quercetin-3-*O*-rutinoside, sophorin and vitamin P [55].

Rutin, like other polyphenolic compounds is found in several fruits and vegetables such as apple peels, black tea, asparagus, buckwheat, onions, green tea, figs, and most citrus fruit like grapefruit, lemon, lime, apple, berries (mulberry), ash tree fruits, and cranberries. Rutin was also found in several African medical plants such as *Araliaceae*, *Moringaceae*, *Leguminosae*, *Guttiferae*, *Euphorbiaceae*, and *Convolvulaceae* [56]. Rutin has demonstrated a number of beneficial activities, including antioxidant, anti-inflammatory, cytoprotective, vasoprotective, anticarcinogenic, neuroprotective, cardioprotective, antiasthmatic, antimycobacterial, antifungal and antiviral activities [57]. In fact, rutin exerts its main activities from its ability to inhibit the nuclear factor NFκB, ERK1/2 up-regulation of Nrf which leads to reduced pro-inflammatory agents such as IL-6, TNF-α, VCAM-1, ICAM-1, and E-selectin [58]. A combination of rutin and chloroquine successfully discloses an antimalarial activity in white leghorn chickens infected with Plasmodium (Bennettinia) juxtanucleare [59]. Sodium rutin sulfate, a sulfated rutin modified from the natural flavonol glycoside rutin was revealed in vitro to possess an antiretroviral effect against HIV-1 X 4 viruses IIIB, HIV-1 R5 isolates Ada-M and Ba-L strains by blocking viral entry and virus-cell fusion through interacting with the HIV-1 envelope glycoprotein [60]. Methanolic extract of *Capparissinaica Veill*, which consists mainly of rutin, was tested for its in vitro antiviral activity against avian influenza strain H5N1 using plaque inhibition assay in Madin-Darby canine kidney. The test showed a significant inhibition of the virus by about 73% [61]. Moreover, rutin, isolated from *Prunus domestica* was suggested as a strong inhibitor of hepatitis C virus (HCV) entry; rutin inhibits the early entry stage of HCV lifecycle by directly acting on the viral particle [62]. Based on the above summarized effects of rutin, this flavonoid appears to be a potent component that could be considered in the treatment of several viral infectious diseases.

## Resveratrol

**Fig. 4:**
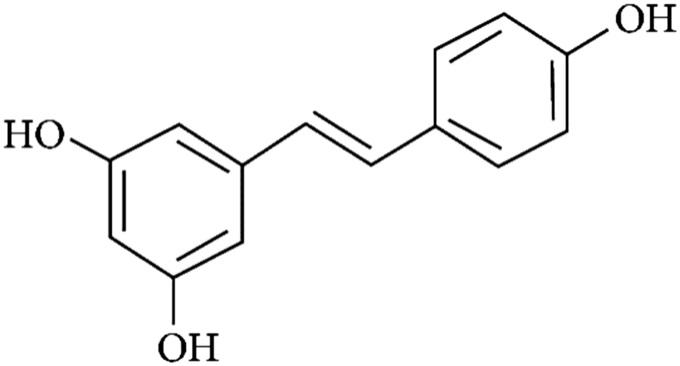
Structure of resveratrol (3,5,4′-trihydroxy-trans-stilbene)

Resveratrol (3,5,4′-trihydroxy-trans-stilbene) (Fig. 4), a natural polyphenol non-flavonoid compound produced by several plants in response to injury or invasion by pathogens, such as bacteria and fungi [63].

Resveratrol is mainly present in pigments of several fruits and vegetables, for instance rhubarb, blueberries, many red grapes, raspberries, mulberries, peanuts and also in dark chocolate and red wine. Resveratrol received intensive studies due to its diverse biological activities, including, antioxidant, anti-inflammatory, antitumor, cardio protective, and antiviral activity, it also possesses other properties such as a preventive and defensive mean towards obesity, diabetes and metabolic syndrome; it can also beneficially regulate the immune system [64, 65]. Resveratrol can affect several pathways, which regulate inflammation, immune response, and cellular response to a variety of stimuli [66]. In fact, resveratrol has become a household word in the context of a natural product that has the potential of promoting good health [67]. In fact, several RNA virus replications have been shown to be inhibited by resveratrol including the influenza virus and rhinoviruses, which are, the main cause of common colds [68]. Resveratrol could be used to prevent respiratory infections in children by a significant reduction in nasal obstruction, rhinorrhea, sneezing, cough, fever and medication usage [69]. Respiratory syncytial virus (RSV) which is the main cause of severe, lower respiratory tract infections in infants was found to be improved by resveratrol intake which reduces IFN-γ levels associated with RSV-mediated airway inflammation [70]. Moreover, resveratrol was found to be a potential antiviral agent in vitro against MERS-CoV infection [71]. Moreover, stilbene derivatives to which resveratrol belongs were shown to totally inhibit the antiviral activities of SARS-CoV [72]. Due to the numerous beneficial effects of resveratrol and its ability to interact with several cellular pathways, it could be selected as a potential candidate for treatment of COVID-19 infection and its related symptoms.

## Kaempferol

**Fig. 5:**
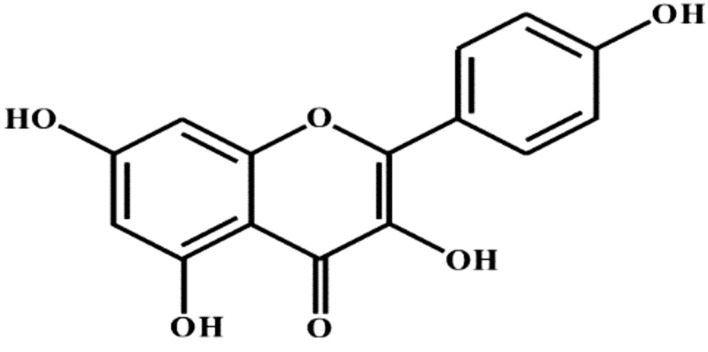
Structure of kaempferol (3,4′,5,7-tetrahydroxyflavone)

Kaempferol also named 3,4′,5,7-tetrahydroxyflavone (Fig. 5) is a widespread natural flavonoids found in a variety of medicinal plants, fruits, vegetables and herbs including grapes, apples, strawberries, tomatoes, broccoli, tea, spinach, beans, kale, leeks, ginkgo biloba leaves, *Tilia spp*, *Equisetum spp*, *Moringa oleifera*, *Sophora japonica* and propolis [73, 74].

Kaempferol has a wide variety of pharmacological activities including antioxidant, anti-inflammatory, antimicrobial, anticancer, cardioprotective, neuroprotective, antidiabetic, anti-osteoporotic, estrogenic/antiestrogenic, anxiolytic, analgesic, and antiallergic activities [75]. In fact, many studies demonstrated the beneficial effects of dietary kaempferol in reducing the risk of chronic diseases by augmenting the body’s antioxidant defense against free radicals, which reinforces body immunity [73]. Kaempferol with its strong anti-inflammatory, anti-oxidant properties decreased lipopolysaccharide (LPS)-induced TNF-α and IL-1β expression by increasing the number of activated macrophages and inhibited NF-κB translocation to the nucleus, which blocks the inflammatory cascade pathway [76]. Kaempferol was established to have a high antiviral activity on both influenza viruses, H1N1 and H9N2 where kaempferol acts on the virus neuraminidase protein and its specific functional groups [77]. Kaempferol is considered as an effective drug in vitro and in vivo for the potential treatment of H9N2 influenza virus-induced inflammation and lung injury. Kaempferol treatment attenuated pulmonary edema, pulmonary capillary permeability, myeloperoxidase (MPO) activity, and the numbers of inflammatory cells. Kaempferol reduced ROS and Malondialdehyde (MDA). In addition, kaempferol also reduced of ROS, MDA, TNF-α, IL-1β and IL-6 production and significantly inhibited the upregulation of toll-like receptor 4 (TLR4), myeloid differentiation factor 88 (MyD88) and phosphorylation of NF-κB and MAPKs pathways [78]. Moreover, kaempferol showed an antiviral activity against influenza A replication and its spread through modulating cell-autonomous immunity through MAPK signaling pathways [19]. Kaempferol derivatives such as kaempferol glycosides and acylated kaempferol glucoside showed comparable antiviral activities against the 3a channel protein of SARS coronavirus that may become expressed in the infected cells. Therefore, inhibition of virus production and allowing the infected body to build up or strengthening its own immune system [79]. Likewise, a docking study indicated that kaempferol derivatives effectively reduced the proteolytic activity of Middle East respiratory syndrome‐coronavirus (MERS‐CoV) by its ability to occupy the S1 and S2 sites of MERS‐CoV 3CLpro [9]. Therefore, Kaempferol and its derivatives might be selected as alternatives to fight COVID‐19 infection possibly through targeting coronavirus proteases.

## Apigenin

**Fig. 6:**
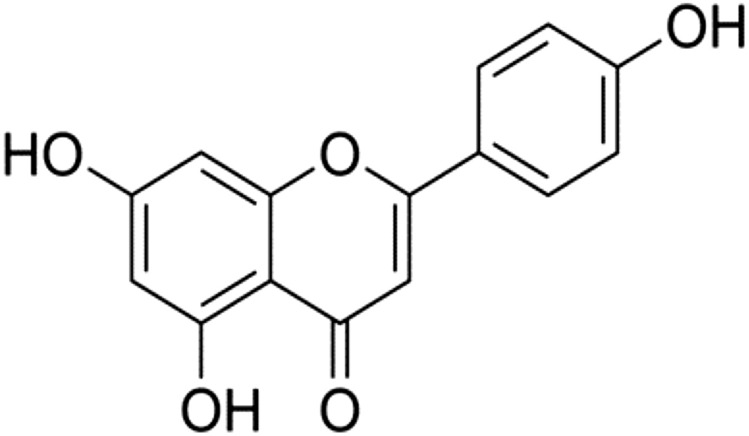
Structure of apigenin (4′,5,7-trihydroxyflavone)

Apigenin (4′,5,7-trihydroxyflavone) (Fig. 6), a naturally occurring flavone compound is found in large quantities in fruits, vegetables, beverages and medicinal herbs such as parsley, grapes, apples, celery, celeriac, thyme, oregano, basil, chamomile tea, beer, and wine [80].

Apigenin encompasses a number of biological functions, for instance strong anti-inflammatory, antioxidant, antibacterial and antiviral activities and blood pressure reduction agent. Several in vivo and in vitro studies and clinical trials suggested that apigenin is a potent therapeutic agent to overcome diseases such as rheumatoid arthritis, autoimmune disorders, Parkinson’s disease, Alzheimer’s disease, and a various type of cancers [81, 82]. In fact, apigenin stimulates different anti-inflammatory pathways, including p38/MAPK and PI3K/Akt, prevents the nuclear translocation of the NF-κB, reduces COX-2 activity and strongly decreases levels of IL-6, TNF-α and IFN-γ levels [80]. Moreover, the influenza virus H3N2 was found to be inhibited by *Elsholtzia rugulosa* (*Lamiaceae*), a common Chinese herb contains apigenin and luteolin among other flavonoids [83]. Furthermore, apigenin has shown several antiviral activities against different viruses such as adenoviruses (ADV) and hepatitis B virus in vitro, African swine fever virus (ASFV), by suppressing the viral protein synthesis and reducing the ASFV yield by 3 log, inhibition of viral protein synthesis through suppressing viral IRES activity of picornaviruses and disrupting viral RNA of enterovirus-71 (EV71) [84]. An anti-inflammatory effect of apigenin-7-glycoside in LPS-stimulated acute lung injury via downregulation of oxidative enzyme expression and protein activation through inhibition of MAPK phosphorylation and NF-κB pathways was also reported which may be a promising therapeutic candidate for various lung inflammatory disorders, such as lung disease and obstructive pulmonary [85]. Apigenin among other flavonoids has been reported to inhibit the proteolytic activity of SARS-CoV 3CLpro. The antiviral effect is presumed to be directly linked to suppress the activity of SARS-CoV 3CLpro [40] and thus apigenin intake, through either a regular diet or supplements, may be beneficial for chronically infected disease such as COVID19.

## Luteolin

**Fig. 7:**
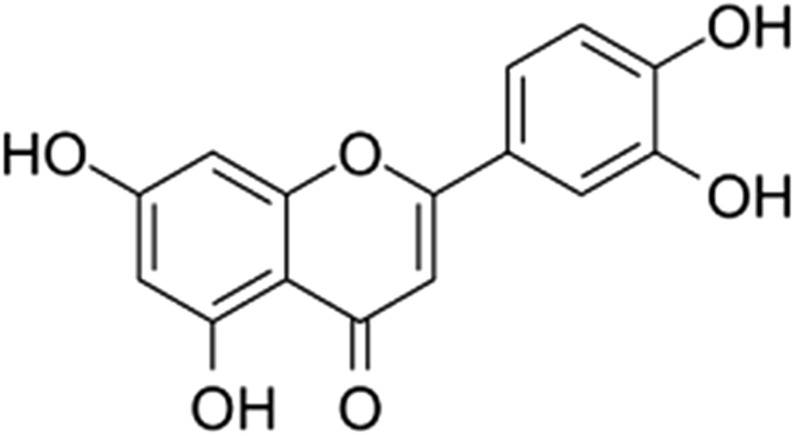
Structure of luteolin (3′,4′,5,7-tetrahydroxyflavone)

Luteolin, 3′,4′,5,7-tetrahydroxyflavone (Fig. 7) which belongs to the flavone group of flavonoids, is a natural yellow dye found widely in the plant kingdom including fruits, vegetables, and medicinal herbs such as broccoli, pepper, thyme, and celery, carrots, olive oil, peppermint, thyme, rosemary, oregano, rosemary, navel oranges, dandelion, perilla leaf, chamomile tea [86].

Luteolin, like some other flavonoid compounds exerts several pharmacological activities, immuno-modulating, anti-inflammatory, anticancer, antimicrobial, antiviral, anti-oxidant, anti-allergic and neuroprotective activities [86, 87]. Luteolin suppressed LPS-elicited inflammatory events in mouse alveolar macrophages including COX2, the secretion of the pro-inflammatory agents, TNF-α, IL-6 and iNOS and ROS production was blocked by repressing NF-κB and AP-1 activation pathways, suggesting a possible therapeutic application of luteolin for treating lung inflammatory disorders [88]. A protection mechanism of luteolin against acute lung injury induced by lipopolysaccharide in mice was suggested by Akt/NFκB inhibition pathway [89]. Similar mechanism of luteion protection activity against lipopolysaccharide-induced acute lung injury and respiratory burst involved inhibition of MEK/ERK and PI3K/Akt pathways was suggested in neutrophils [90]. Moreover, luteolin inhibited Epstein-Barr virus (EBV) reactivation by repressing the immediate-early genes Zta (Zp) and Rta (Rp) and also inhibited Sp1-luc activity in the virus promoters, suggesting luteolin is a potential dietary compound for prevention of virus infection [91]. Furthermore, luteolin has been found to be a potent antifibrotic activity as it inhibits lung inflammation and suppresses of myofibroblast differentiation as well as epithelial-to-mesenchymal transition [92]. Anti-asthmatic activity of luteolin was also reported in experimental mice [93]. In addition, two small molecules, tetra-*O*-galloyl-β-d-glucose (TGG) and luteolin, were identified, whose anti-SARS-CoV activities were confirmed using a wild-type SARS-CoV infection system. Both TGG and luteolin showed at the same concentration levels to effectively inhibit the entry of HIV-luc/SARS pseudo typed virus into its host, which suggests a potential clinical use of these two molecules as anti-SARS drugs [41, 94]. Consequently, luteolin could be selected as an alternative treatment or body immunity enhancer against COVID‐19 infection due to its strong anti-viral and anti-inflammatory properties during infection.

## Genistein

**Fig. 8:**
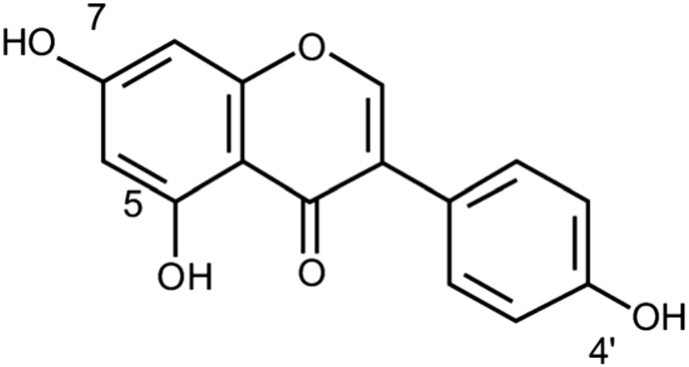
Structure of genistein (4′,5,7-trihydroxyflavone)

Genistein (4′,5,7-trihydroxyflavone) (Fig. 8), is a naturally occurring phytoestrogen found mainly in soy foods and legumes including peas, lentils or beans, fava beans, lupin, tofu, kudzu, psoralea, red clover, coffee and some medical plants such as *Flemingia vestita* and *Flemingia macrophylla* root, [96].

Genistein possesses many therapeutic uses against numerous disorders including osteoporosis, cardiovascular diseases, a variety of tumors, diabetes, inflammation, oxidative stress and metabolic syndromes. Genistein was successfully used as an immune-suppressive agent both in vitro and in vivo [97] due to its ability to inhibit the infectivity of enveloped or non-enveloped viruses, as well as single or double-stranded RNA or DNA viruses by different mechanisms. Genistein has been shown to reduce the infectivity of a variety of viruses affecting humans and animals, including adenovirus, herpes simplex virus, human immunodeficiency virus, porcine reproductive and respiratory syndrome virus, and rotavirus [98]. An ethyl acetate extract from Chongkukjang, a traditional Korean fermented product prepared from soybeans, was shown as an effective therapeutic agent to treat influenza A virus infection [99]. Moreover, genistein was also effective against the ionic channel of HIV, which is supposed to form a cation-permeable ion channel in the infected cell [105]. Genistein may be also a useful candidate for developing a new anti-rotavirus (RV) by inhibiting rotavirus replication and upregulating aquaporin 4 (AQP4) expression via the cAMP/PKA/CREB signaling pathway [100]. A study has reported that an increasing consumption of genistein or a diet with moderate to high amounts of soy genistein is associated with a better lung function in patients with asthma [101]. The African swine fever virus (ASFV) was inhibited in vitro by genistein through disrupting the viral DNA replication, blocking the transcription of late viral genes as well as the synthesis of late viral proteins [102]. It has been demonstrated that pre-treatment of host cells with the kinase inhibitors (genistein and tyrphostin) leads to an inhibition of infection or transduction in cells infected with Ebola virus, Marburg virus, and Lassa virus [103]. Therefore, the strong influence of genistein as an antiviral agent against different kinds of virus infections may promote its use as a potential candidate for defeating COVID-19 and related symptoms.

## Naringenin

**Fig. 9:**
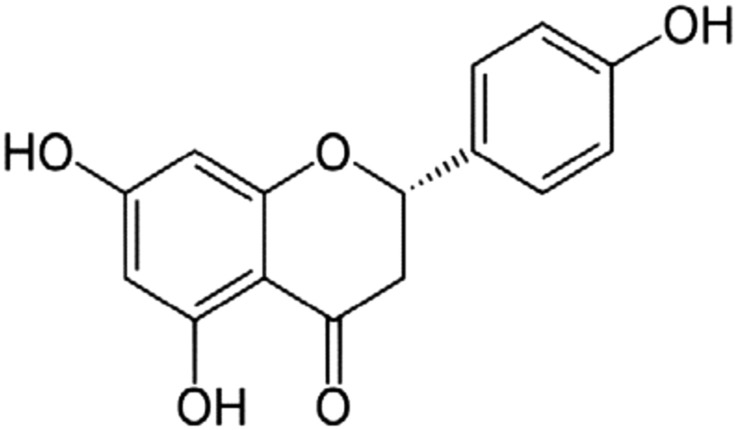
Structure of naringenin (4′,5,7-trihydroxytlavanone)

Naringenin (4′,5,7-trihydroxytlavanone) (Fig. 9) occurs naturally in a variety of citrus fruits, bergamot, tomatoes, tomato food products, grapefruit, sour orange, cherries, cocoa and it is may be found in some herbs such as oregano and water mint. Naringenin has many pharmacological effects, including antidiabetic, antiatherogenic, antidepressant, immunomodulatory, antitumor, antiinflammatory, DNA protective, hypolipidaemic, antioxidant, antiasthma, antiviral, antibacterial and peroxisome proliferator-activated receptors (PPARs) activator [56, 104].

Naringenin blocks MAPKs phosphorylation by decreasing translocation and DNA binding of NF-κB and AP-1, which restrain the production of proinflammatory cytokines such as IL-33, TNF-α, IL-1β, and IL-6 [105]. It has been reported that naringenin suppressed respiratory overexpression and eosinophilic airway inflammation in asthma and thus, reduced acute neutrophilic airway inflammation by blocking the NF-κB pathway [106]. Naringenin has been found to impair replication of several viruses in human cells such as dengue, Zika and Chikungunya viruses [107–109] and to guarantee a significant protection against LPS-induced acute lung injuries through its anti-inflammatory, antioxidant, antinitrosative and antiapoptotic effects [110, 111]. Due to its strong anti-inflammatory and anti-oxidant effects, narigenin may be utilized against pneumonia companied with the spread of COVID-19.

## Gallic acid

**Fig. 10:**
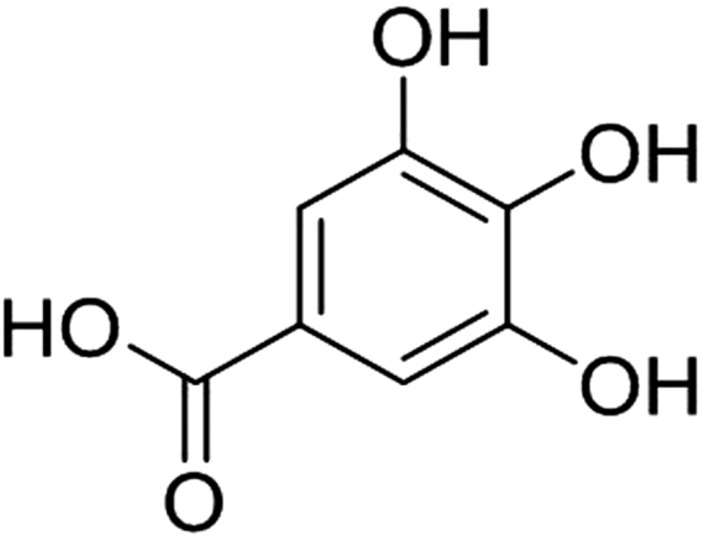
Structure of gallic acid (3,4,5-trihydroxybenzoic acid)

Gallic acid (3,4,5-trihydroxybenzoic acid) (Fig. 10), a compound found in several fruits and medicinal plants including gallnuts, grapes, tea, hops, oak bark, sumac, black raspberry, witch hazel and mostly in certain red fruits, black radish, and onions is a phenolic acid that comprises several valuable pharmaceutical properties including antioxidant, anti-inflammatory, antineoplastic, anti-cancer, gastrointestinal and cardiovascular protective, antiviral, antibiotic and antimicrobial.

The strong anti-oxidant capacity of gallic acid grants it an important role in absorbing and neutralizing free radicals, with even better results than some vitamins [112, 113]. It was reported that, gallic acid has several antiviral activities such as anti‐enterovirus 71 (EV71), anti-herpes simplex virus (HSV)-2 and anti‐human immunodeficiency virus activity and anti-hepatitis C (HCV) [114, 115]. Gallic acid of black raspberry seeds was demonstrated in vitro to have an antiviral activity against influenza A and B type viruses which suggested a promising role in targeting virus particles [116]. Similarly, anti-pandemic potential role of gallic acid against influenza A (H1N1) virus was demonstrated through the down-regulation of adhesion molecules and chemokine to prevent viral attachment and inhibition of the virus mRNA replication [117]. A study has reported that inhibition of human rhinoviruses (HRV) production by gallic acid is mainly due to its general role as an antioxidant and the mode of action derived from the inhibition of virus absorption [118]. Moreover, gallic acid along with other related phenolic acids (caffeic acid, chlorogenic acid) was found to exert sustainable anti-viral activity against human coronavirus NL63 (HCoV-NL63), one of the main circulating coronaviruses worldwide that causes respiratory tract diseases like runny nose, cough, bronchiolitis and pneumonia [119]. Based on the above information, gallic acid could be proposed as an alternative treatment for COVID-19 and its related symptoms.

## Caffeic acid

**Fig. 11:**
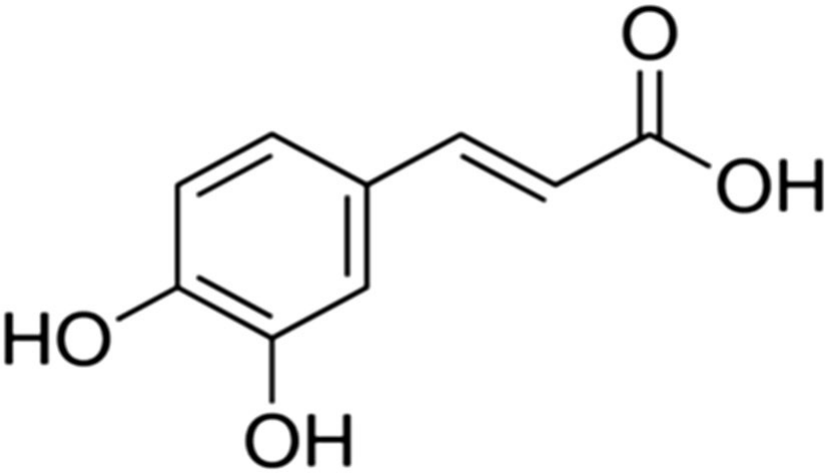
Structure of caffeic acid (3,4-dihydroxycinnamicacid)

Caffeic acid (3,4-dihydroxycinnamicacid) (Fig. 11) is one of the most common phenolic acids, which occurs frequently in fruits, grains, medicinal herbs and dietary supplements. [120, 121].

In vitro and in vivo experiments showed that caffeic acid and its derivatives such as caffeic acid phenethyl ester have numerous physiological activities including antibacterial, antiviral, antioxidant, anti-inflammatory, anti-atherosclerotic, immunostimulatory, antidiabetic, cardioprotective, antiproliferative, hepatoprotective, anticancer, and anti-hepatocellular carcinoma activity [121, 122]. Caffeic acid of *Sambucus Formosana Nakai* ethanol extract significantly repressed the replication of human coronavirus NL63 (HCoV-NL63) that causes upper respiratory tract illnesses, in a cell-type independent manner, and blocked virus attachment. The study proposed that caffeic acid could be the vital component with anti-HCoV-NL63 activity [119]. In vitro caffeic acid demonstrated an antiviral activity by acting on severe fever with thrombocytopenia syndrome (SFTS) virus, which causes tick-borne hemorrhagic fever in East Asia, and hinders viral infection and spread by inhibiting the binding of SFTSV to the cells [123]. Conjugation of peramivir, an influenza inhibitor with caffeic acid has showed a better drug efficacy against influenza and was found to inhibit influenza neuraminidase [124, 125]. Moreover, a study has reported antiviral activity of caffeic acid towards herpes simplex (HSV), VSV-Ebola pseudotyped and vaccinia viruses and that antiviral activity increased and occurred early in the virus replication cycle with the addition of chelated inorganic ions or a metal such as iron to caffeic acid [126]. Caffeic acid and its derivatives have uncountable antiviral activities that may be used against the spread of emerging COVID-19 and its related symptoms.

## Daidzein

**Fig. 12:**
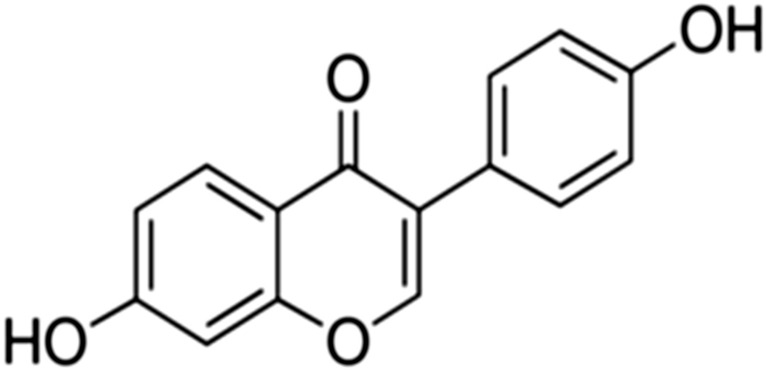
Structure of daidzein (4′,7-dihydroxyisoflavone)

Daidzein (4′,7-dihydroxyisoflavone) (Fig. 12) is a naturally occurring isoflavone compound found in high concentrations in soybeans, soy products like tofu and textured vegetable protein, red clover and also in some herbs such as Vitex Agnus Castus and Black Cohosh. It is also present in many other vegetables, fruits, nuts, peas, lentils, various cereals, bakery products, milk, meat and other food products [127, 128].

Daidzein is classified as a phytoestrogen because it has oestrogen-like properties; with its partner genistein, daidzein compromises 90% of the intake of oestrogenic isofalvones from the diet [127, 129]. Daidzein has several biological and pharmacological properties for instance antioxidant, anticancer, anti-inflammatory, neuroprotective, protective treatment of cardiovascular diseases, and autoimmune diseases [130]. Soy isoflavones consumption such as genistein, daidzein, and glycitein has been associated with a reduced prevalence of chronic health disorders and regulation of the immune response, which give them an important potential role in clinical applications in immune-dysfunction [131]. Daidzein markedly attenuated TNF-α-induced lung inflammation and lipopolysaccharide-induced acute lung injury through blocking of NF-κB pathway via different interaction mechanisms, which inhibited several pro-inflammatory markers such as Cxcl2 in lung tissues [132]. Moreover, a study has reported that daidzein exhibits an anti-fibrotic effect against Bleomycin (BLM) induced pulmonary fibrosis (PF) in rats [133]. Furthermore, during influenza infection, reactive oxygen species (ROS) and lipid peroxides are generated in several tissues such as the lung, and daidzein was reported to exert an antiviral activity through its antioxidant capacity to decease ROS and lipid peroxide generations. Consequently, daidzein regulates influenza virus replication via signal transduction through 5-lipoxygenase products induced by daidzein [134]. Also, novel daidzein analog compounds synthesized based on structural modifications of daidzein have been demonstrated in vitro a selective antiviral agent against H1N1 TR influenza viruses [135]. Due to their role in the regulation of immune responses and antioxidant activity, daidzein could be applied to enhance body immunity against emergence diseases such as COVID-19 and related immunity dysfunctions.

## Chrysin

**Fig. 13:**
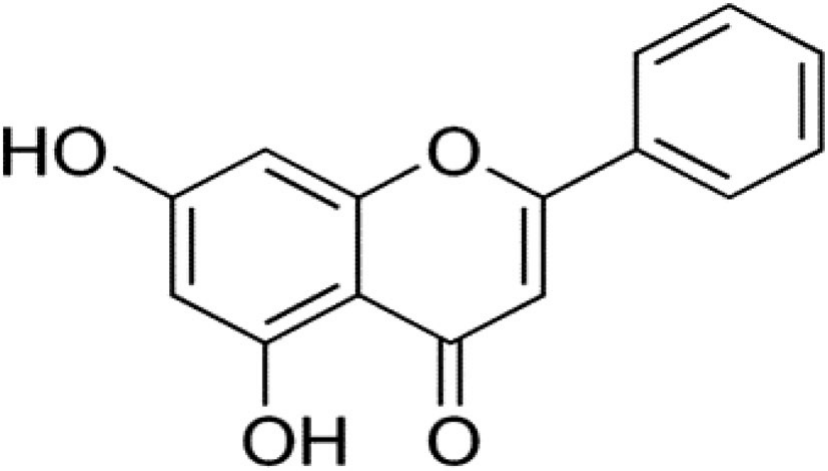
Structure of chrysin (5,7-dihydroxyflavone)

Chrysin (5,7-dihydroxyflavone, also known as vitamin P) (Fig. 13) is a bioflavonoid compound found in high concentrations in propolis, honeym passion fruit, flowers, mushrooms, carrot, chamomile and some medicinal plants such as *Radix scutellariae*, *Lactariusdeliciosus* (*L. ex Fr.)S.F. Gray*, *Passiflora incarnate L.*, *S. ramosissima M. Pop*, *Cytisusmultiflorus* (*L’Her. Ex Aiton*) *Sweet*, *Scutellaria immaculate Nevski ex Juz*, and *Passifloracoerulea L*. *Desmoscochinchinensis Lour*.

Chrysin exhibits many biological activities and pharmacological effects, including antioxidant, anti-inflammatory, anticancer, pro-apoptotic, antiangiogenic, antimetastatic, immunomodulatory, antiasthmatic, antibacterial and antiviral activities [136, 137]. Actually, chrysin exerts its activities against various diseases by different mechanisms including the suppression of inducible nitric oxide synthase (iNOS) and NF-κB, inhibition of histone deacetylase and DNA topoisomerases, inhibition pro-inflammatory cytokine expressions such as TNF-α and IL-1β through blocking histamine release and also decreasing matrix metalloproteinase-2 (MMP-2) expression [138]. Chrysin could also prevent airway inflammation by inhibiting the release of pro-inflammatory mediators TNF-α, IL-1β, IL-8, and myeloperoxidase (MOP) expression via restraining ERK and P38 pathway [136, 138]. Chrysin has been shown to be a potent inhibitor of human immunodeficiency virus (HIV) and also has been shown to inhibit the enterovirus A71 (EV-A71) replication [139, 140]. Moreover, it has been reported that chrysin inhibited the interaction between SARS-CoV spike (S) protein and angiotensin-converting enzyme 2 (ACE2) which is the functional receptor for SARS-CoV [52, 141]. Consequently, this huge capacity of chrysin in protecting against several diseases especially virus infections may give it a vital role in the fight against COVID-19 principally, via its ability to interact with the virus receptor.

## Ferulic acid

**Fig. 14:**
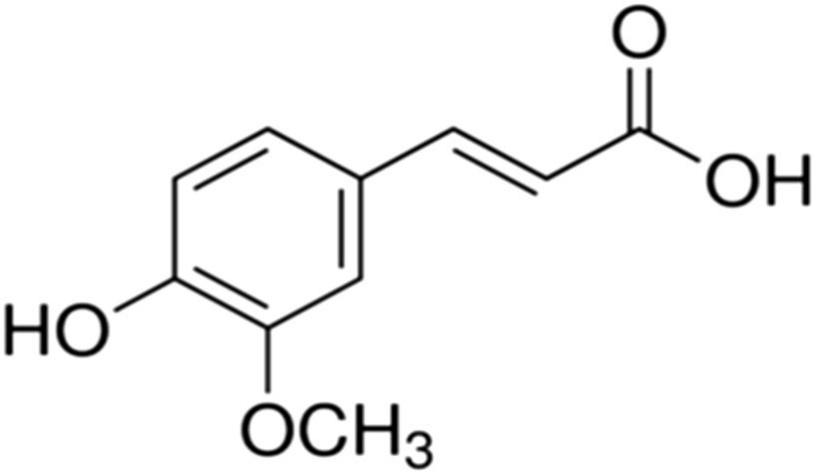
Structure of ferulic acid (4-hydroxy-3-methoxycinnamicacid)

Ferulic acid (4-hydroxy-3-methoxycinnamicacid) (Fig. 14) is a phenolic acid generally found in plant cell walls such as whole grains, spinach, parsley, grapes, rhubarb, coffee and cereal seeds, mainly wheat, oats, rye, rice barley, pineapple, maize bran, some fruits such as eggplant, orange and grapefruit, vegetables such as tomato and carrot, flax, beets, broccoli and sweet corn [142, 143].

Ferulic acid is rapidly absorbed into the body and stays in the blood stream longer than any other antioxidant, even longer than vitamin C. Because of these features, ferulic acid is considered to be a superior antioxidant and therefore it is widely used in health foods and nutrition [144]. In addition, ferulic acid possesses many physiological activities such as anti-inflammatory, antimicrobial, antiviral, anticancer, antiarrhythmic, antithrombotic, antidiabetic, lipid peroxidation prevention, free radical scavenger; increase NO synthesis and immunostimulant properties [142–144]. Sodium ferulate, the sodium salt of ferulic acid, in a combination with oxymatrine had a protective effect on LPS-induced acute lung injury in mice principally by inhibiting the production of CRP and TNF-α, which inhibited the myeloperoxidase (MPO) activity in lung homogenate and attenuated inflammatory cell numbers and protein concentration in the bronchoalveolar lavage fluid. Similarly, sodium ferulate protects against influenza virus infection through activation of the TLR7/9-MyD88-IRF7 pathway, which recognizes viral nucleic acids and activates different cascades that contribute to the production of interferons (IFNs) and also by inhibiting the NF-κB pathway, which resulted in blocking the influenza virus replication [145, 146]. Moreover, a ferulic acid derivative had the best inhibition activity against the neuraminidase (NA) influenza virus (H1N1), in vitro, due to the interactions with conserved and essential residues of NA, which is comparable to those of oseltamivir and zanamivir, two available commercial NA inhibitors [147]. The presented properties of ferulic acid and its derivatives sodium ferulate solely or in a combination with other compounds may warrant their use in the fight against such emergence disease similar to COVID-19.

## Hesperetin

**Fig. 15:**
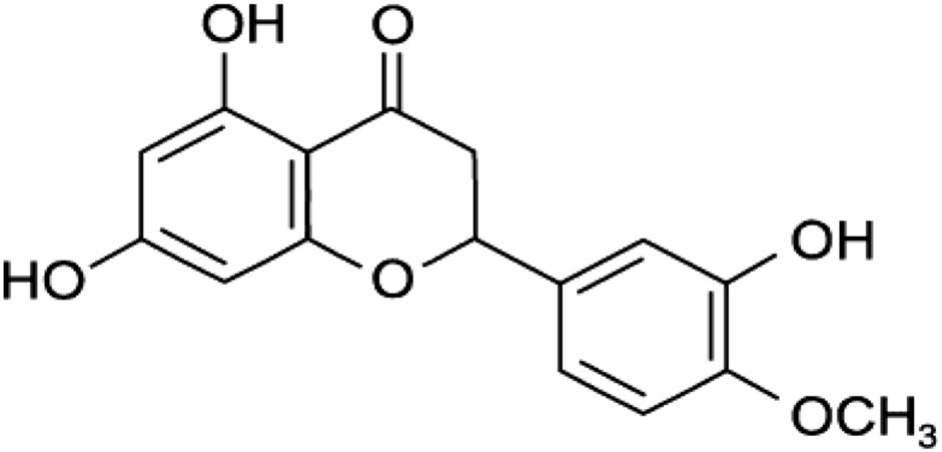
Structure of hesperetin (3′,5,7-Trihydroxy-4′-methoxyflavanone)

Hesperetin (3′,5,7-trihydroxy-4′-methoxyflavanone) (Fig. 15) a naturally occurring flavone is found predominantly in citrus fruits, lemons, sweet oranges, bitter orange, and grapefruit juices. In addition, it occurs in a large number of fruits and vegetables such as tomatoes and cherries, and various herbal formulations [148].

Hesperetin and its metabolites display several biological activities including antioxidant, anti-inflammatory, and lipid decreasing levels, anticarcinogenic, antidiabetic, immunoregulatory, and neuroprotective [149, 150]. Actually, several in vitro and in vivo studies have been reported that Hesperetin, its metabolites, and its synthetic derivatives augmented the antioxidant cellular defenses such as the ERK/Nrf2 signaling pathway and peroxisome proliferator-activated receptor-γ (PPAR-γ), reduced inflammatory targets including NF-κB, iNOS, and COX-2, and other markers of chronic inflammation such as IL-1β, IL-6, NO and TNF-α [151–153]. Hesperetin has been shown a potential drug for acute lung injury (ALI) induced in vivo by lipopolysaccharide by downregulated the Toll-like receptor 4 (TLR4) and suppressed NF-κB activation in lung tissue [154]. Hespertein plus naringenin has a protective capacity against lung fibrosis by reducing airway inflammation in murine chronic asthma model. In addition, the anti-fibrotic effects of hespertein have been indicated in the liver and kidney [155, 156]. Hespertin exerted inhibitory effect on the intracellular replication of transmitted Chikungunya virus (CHIKV) and exhibits drug-like properties which maybe a potential as a therapeutic option for CHIKV infection [157]. Moreover, hesperetin has shown an antiviral activity against the human Respiratory Syncytial Virus (hRSV) trough binding to M2-1 virus protein, which is an important transcriptional anti-termination factor and a potential target for viral replication inhibitor development [158]. Among several compounds that exhibit an in vitro activity against SARS-CoV, hesperetin, was the most selective with a selectivity index of ∼300. Hesperetin dose-dependently suppressed the cleavage activity of the 3C-like protease (3CLpro) of SARS-CoV in cell-free and cell-based assays [159]. Furthermore, a recent study has reported that hesperetin has the potential to inhibit angiotensin-converting enzyme 2 (ACE2), the same host receptor of SARS-CoV and thus block infection with SARS-CoV-2 [160]. Therefore, these studies clearly suggest that hesperetin could play a key role in the prevention and treatment of COVID-19 and related pneumonia.

## Vanillic acid

**Fig. 16:**
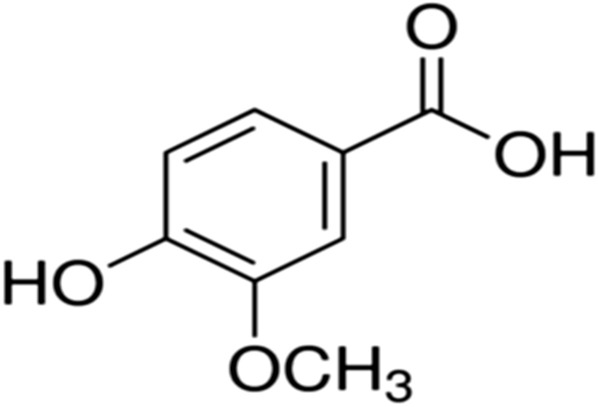
Structure of vanillic acid (4-hydroxy-3-methoxybenzoicacid)

Vanillic acid (4-hydroxy-3-methoxybenzoicacid) (Fig. 16), an intermediate in the production of vanillin from ferulic acid is the main flavor component of cured vanilla beans. Vanillic acid is found in the roots of *Angelica sinensis*, a plant used in traditional Chinese medicine, açaí oil, olive oil vinegar and wine [161, 162].

Vanillic acid is a well-known generally regarded as a safe flavoring agent with beneficial biological activities such as anti-oxidant, anti-lipid peroxidative, anti-inflammatory, anti-apoptosis, neuroprotective/cognitive and regulation of insulin secretion [163, 164]. Several studies have reported the efficiency of vanillic acid in controlling the immune response or the inflammatory ones. Vanillic acid improved the activity of human lymphocyte proliferation and secretion of interferon-gamma (INFγ) in human peripheral blood mononuclear cells. Additional study indicated that vanillic acid has a hepatoprotective effect through its suppressive action on immune-mediated liver inflammation in concanavalin A-induced liver injury [165, 166]. Vanillic acid was found to inhibit inflammatory state in mice by suppressing neutrophil recruitment and its mechanisms of action involves antioxidant effects and NFκB-related inhibition of pro-inflammatory cytokine production such as IL6, COX2, IL-1β and TNF-α [167, 168]. Moreover, the major compounds extracted from the root of *Rubia cordifolia* (xanthopurpurin and vanillic acid), traditionally used as a hemostatic agent inhibited effectively rotavirus multiplication by promoting virus-induced apoptosis in MA-104 cells [169]. Consequently, vanillic acid may have a role in the combat against pneumonia related COVID-19 infection.

## Galangin

**Fig. 17:**
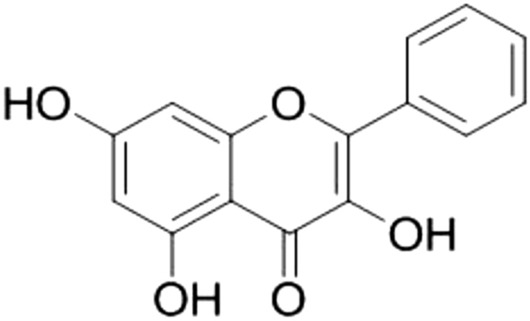
Structure of galangin (3,5,7-trihrdroxyflavone)

Galangin (3,5,7-trihydroxyflavone) (Fig. 17), is a flavonoid compound found in high concentrations in *Alpinia officinarum* Hance (lesser galangal), which has been used as an herbal medicine for colds, stomachaches, and swellings, or as a food additive in Asia. Galangin, which has been used as a cure for many symptoms, particularly in China is also found in honey, propolis and *Helichrysum aureonitens* [170].

Several epidemiological studies in vitro and animal studies have claimed that galangin and the consumption of galangin-containing foods may affect several diseases [171]. Galangin has a broad range of biological properties, including anti-oxidative and free radical scavenging, modulating enzyme activities, suppressing chemical genotoxicity, inhibitory effects on various microbes, the control of hypertension, diabetes, and chemoprevention of several cancers anti-inflammatory and anti-fibrotic activities in various disorders [172–174]. Galangin attenuated in vitro and in vivo airway remodeling by inhibiting TGF-β1-mediated ROS generation and MAPK/Akt phosphorylation in asthma; also, galangin improved ovalbumin (OVA)-induced airway inflammation by inhibiting NF-κB pathway, which reduced eosinophils, neutrophils, and lymphocytes infiltration and goblet cell hyperplasia. In addition, galangin reduced expression of iNOS, VCAM-1, TNFα induced p65 nuclear translocation, expression of monocyte chemoattractant protein-1 (MCP-1), eotaxin, and C-X-C motif chemokine 10 (CXCL10) levels in lung tissue [175, 176]. In addition, galangin reduced LPS-induced acute lung injury by inhibition of inflammation and oxidative stress. In fact, protective effects of galangin were associated with inhibition of NF-κB and upregulation of heme oxygenase (HO)-1. Similarly, galangin exerted profound anti-asthmatic property by activating PPARγ, which resulted in galangin alleviating airway inflammation in murine model of asthma. [176–178]. Furthermore, at concentrations ranging from 12 to 47 µg/mL, galangin isolated from the aerial parts of *helichrysum aureonitens* showed a significant antiviral activity against herpes simplex virus type 1 (HSV-1) and coxsackie B virus type 1 (CoxB1) limited activity against reovirus, but no antiviral activity against adenovirus type 31 (Ad31) [179]. The anti-inflammatory and anti-oxidant capacity of galangin may give it a role in the pneumonia associated with COVID-19 and other emergence diseases.

## p-Coumaric acid

**Fig. 18:**
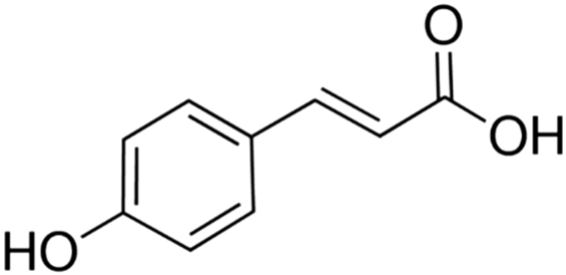
Structure of p-coumaric acid (4-hydroxycinnamicacid)

p-Coumaric acid (4-hydroxycinnamicacid) (Fig. 18), is a phenolic compound and the most frequently occurring isomer of coumaric acid in nature.

p-Coumaric acid classified as a phytochemical and nutraceutical, presents in vegetables, and fruits, such as cranberry syrups, grape juices, tomatoes, apple, peanuts, carrots, garlic, potatoes, onions, beans, and cereals such as rice maize, oats and wheat, tea, beer and chocolate. p-Coumaric acid and its conjugate have demonstrated several biological activities including antifungal, anti-cancer, antimicrobial, anti-viral, anti-melanogenic, antioxidant, immunomodulatory and anti-inflammatory effects, antiplatelet aggregation, anxiolytic, antipyretic, analgesic, anti-arthritis and antigenotoxicity [180–182]. In vitro and in vivo experiments showed that p-coumaric acid attenuated lipopolysaccharide-induced lung inflammation by scavenging ROS production and modulating the oxidative stress under inflammatory conditions in lung injury [180]. Moreover, *Adenostemma lavenia* is a permanent medicinal herb belonging to the Compositae family and is widely distributed in the tropical parts of Asia mainly in Taiwan. It is used to treat pulmonary congestion, pneumonia, bacterial infections of the respiratory tract, edema, and inflammation. p-Coumaric acid was found to be the major constituent of this herb. In fact, *Adenostemma lavenia* has been reported to ameliorate acute lung injury through activating AMPK/Nrf2/HO-1 signaling pathway and improving the anti-oxidant response [183]. p-Coumaric acid among other compounds extracted from the Himalayan medicinal plants traditionally used to treat bronchitis and related symptoms has demonstrated antiviral activities in vitro against different kinds of human rhinoviruses [184]. Likewise, in vitro research showed that p-coumaric acid among other phenolic acid components extracted from propolis could be effective against Herpes Simplex Virus [185]. Furthermore, a recent docking study has reported that cis-p-Coumaric acid found in coriander, the cis-form of p-coumaric acid, among other natural products may interfere with SARS-CoV-2 attachment to the host cell and can be successfully considered as anti-COVID-19 agents for people with a high risk of cell stress like elders, cancer patients, and front-line medical staff. Similarly, another docking study showed that p-Coumaric acid, Curcumin and their boronic acid derivatives are probable inhibitors of endoribonuclease Nsp15 encoded by Middle East Respiratory Syndrome Coronavirus (MERS-CoV) [186, 187]. Consequently, p-Coumaric acid and its derivatives may be used as target for an alternative therapy of covid19 and its related pneumonia.

## Eriodictyol

**Fig. 19:**
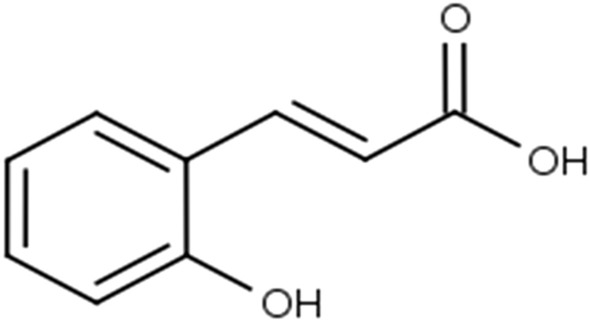
Structure of eriodictyol (5,7,3′,4′-tetrahydroxyflavanone)

Eriodictyol (5,7,3′,4′-tetrahydroxyflavanone) (Fig. 19), is a natural flavonoid isolated mainly from *Lyonia ovalifolia* and *Yerba Santa* (*Eriodictyon californicum*).

Eriodictyol is also detected in some plants of the *Lamiaceae* family, such as peppermint (*Mentha piperita*), oregano (*Origanum vulgare*), and thyme (*Thymus vulgaris*) and it is ubiquitous in fruits, vegetables, including tomatoes, mint, grapefruit, oranges, and lemons as well as in several medicinal plants such as *Bauhinia ungulata*, *Arcytophyllum thymifolium*, *Elsholtzia bodinieri*, and *Clinopodium chinense* [188, 189]. Several studies have revealed that eriodictyol possesses several bioactivities, including anti-inflammation, anti-oxidation, antimicrobial, anticancer, protective effects on the neurons, kidneys, and lungs, insulin secretagogue and antidiabetic properties [190, 191]. Furthermore, several studies have shown that eriodictyol exerts its anti-inflammatory and antioxidant effects through Akt/NF-κB-related signaling pathways [192]. Eriodictyol has immunomodulatory effects, comprising inhibition of nitric oxide (NO) production by blockage of NF-κB activation and phosphorylation of p38, MAPK, extracellular signal-regulated kinase 1 and 2 (ERK1 and 2), JNK and COX-2 in lipopolysaccharide (LPS)-induced inflammatory responses in macrophages [193]. Moreover, a study demonstrated that eriodictyol could alleviate the LPS-induced lung injury in mice by regulating the Nrf2 pathway and inhibiting the expression of inflammatory cytokines in macrophages such as TNF-α, IL-6, IL-1β and MIP-2, suggesting that eriodictyol may be used as a drug for the treatment of LPS-induced lung injury. In addition, its anti-inflammatory and anti-oxidative capacities have drained attention to its therapeutic potential, especially, for asthma and treating colds [194, 195]. A recent study has reported that (2S)-eriodictyol 7-*O*-(6″-*O*-galloyl)-beta-D-glucopyranoside, aneriodictyol derivative, ranked as an inhibitor against SARS-CoV-2 3CLpro receptor binding site and catalytic dyad (Cys-145 and His-41) of SARS-CoV-2 3CLpro [196]. A recent docking study has ranked Eriodictyol among other compounds to be tested experimentally as an inhibitor of ACE2 receptor of COVID-19 [197]. These studies suggested that natural products such as Eriodictyol may prove more useful candidates for COVID-19 drug therapy.

## Syringic acid

**Fig. 20:**
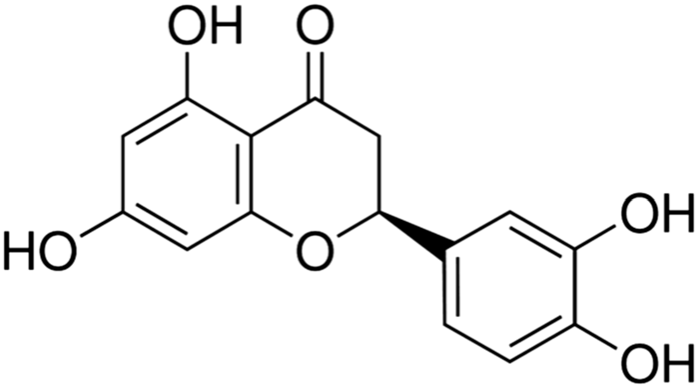
Structure of syringic acid (4-hydroxy-3,5-dimethoxybenzoicacid)

Syringic acid (4-hydroxy-3,5-dimethoxybenzoicacid) (Fig. 20), is a phenolic acid found in many plants and dry fruits such as, dates and walnuts, olives, spices, pumpkin, grapes, acai palm, honey, red wine; cereals and other plants such as gall trees, proso millet, radish, chard and sugar apple [198, 199]. Syringic acid exhibits several positive effects on human health including anti-diabetic, hepato-protective, anti-oxidant, anti-endotoxic, anti-steatotic, anti-inflammatory, anti-hypertensive, neuro-protective, anti-cancer and cardio-protection activities [198–200]. In fact, syringic acid possesses these beneficial effects on human health due to its strong anti-oxidant nature.

Syringic acid exerts its activities via modulation of several molecules involved in disease progression such as proteins, transcriptional factors and growth factors [200]. Syringic acid identified, with other secondary metabolite compounds, in extracts of stems and leaves of *Bougain villeam*, which it is used as a tea for easing cough, fever, sore and diarrhea besides its several beneficial activities such as antioxidant, anticancer, antibacterial, antiviral [201]. A study has reported in asthma mice model that the effect of syringic acid is prominent in the treatment of asthma by controlling the accumulation of inflammatory cells, other inflammatory markers such as IL-4, IL-5, IL-13, and TNF-α along with enhancement of antioxidant markers, suppression of ROS and controlling airway hyper-reactivity. Therefore, syringic acid may be recommended for clinical trials in the treatment of asthma [202]. Moreover, syringic acid has shown among other compounds, to be a potent inhibitor against H1N1 swine influenza [203]. Due to its strong antioxidant capacity, synergic acid, may be used to strengthen lung and body invulnerability in front of COVID-19 infections.

## Polydatin

**Fig. 21:**
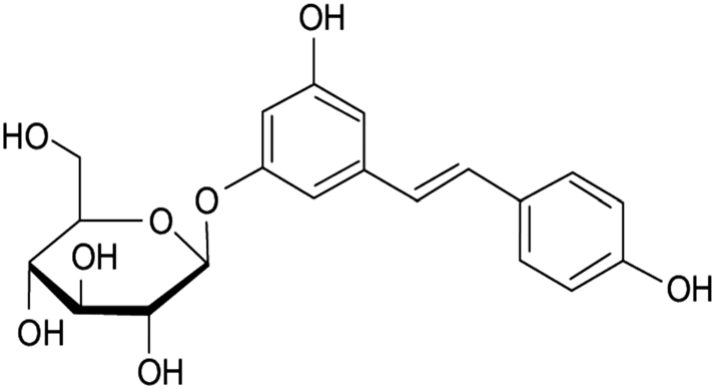
Structure of polydatin (3,4′,5-trihydroxystilbene-3-β-D-glucoside)

Polydatin (3,4′,5-trihydroxystilbene-3-β-D-glucoside) (Fig. 21), also named piceid is a natural resveratrol glucoside and precursor.

Polydatin, an active compound isolated from the root of *Polygonumcus pidatum Sieb. et Zucc*, has been widely used for treatment of hyperlipemia, inflammation, infection and cancer, fever, cough, hypertension and other pharmacological benefits. Polydatin is also detected in grape, peanut, hop cones, red wines, hop pellets, cocoa-containing products, chocolate products and many daily diets [204, 205]. Unlike resveratrol, which passively penetrates cells, polydatin enters cells via an active mechanism using glucose carrier and it is more resistant to enzymatic oxidation than resveratrol and possesses much better solubility in water [206]. Polydatin has numerous benefits which is widely reported including anti‐inflammatory effect in chronic lung diseases, anti-oxidative, anti-platelet aggregative, anti-fibrosis, anti-cancer, benefits for neurological diseases and anxiolytic effects [207, 208]. A study has demonstrated that polydatin improves lipopolysaccharide (LPS)-induced acute respiratory distress syndrome (ARDS). In addition, polydatin has mediated Parkin-dependent mitophagy and protects against mitochondria-dependent apoptosis in ARDS [209, 210] niae (MP) infection, MP can infect both the upper and lower respiratory tracts, was found to be suppressed by polydatin treatment through inhibition of the NACHT domain-, leucine-rich repeat-, and pyd-containing protein 3 inflammasome (NLRP3) and the nuclear factor-κB pathway after MP infection [211]. Actually, several in vivo studies have reported an important protective role of polydatin treatment via different mechanisms in ovalbumin-induced bronchial asthma as well as in LPS-induced acute lung injury [212, 213]. Moreover, polydatin protects the respiratory system from the damage caused by fine particulate matters (aerodynamic diameter < 2.5 μm; PM2.5) during air pollution that may cause deleterious effects such as premature death among individuals due to lung disease, lung dysfunction and asthma exacerbation, mainly by the capacity of polydatin to reduce the injury from oxidative stress and inflammation in lung tissues [214]. Moreover, polydatin can attenuate hypoxic pulmonary hypertension through Protein Kinase C Mechanisms (PKC) and it might be a promising candidate for hypoxic pulmonary treatment [215]. Polydatin was found, among other phenolic compounds extracted from of *Polygonum cuspidatum*, to inhibit influenza A virus replication in A549 cells through toll-like receptor 9-induced (TLR9) interferon beta (IFN-β) expression [216]. A recent docking study has reveal that polydatin, is a potent COVID-19 main protease (Mpro) inhibitor, among other natural phenolic compounds that have been demonstrated to have an anti-coronavirus activity [217]. Therefore, regarding the enormous protective roles of this natural phenolic compound on the respiratory system, it is concluding that polydatin could be consecrated as a potential molecule against COVID-19 infections and its related symptoms.

## Neobavaisoflavone

**Fig. 22:**
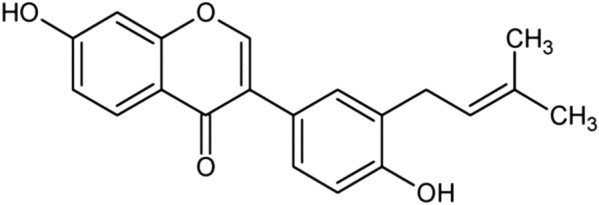
Structure of neobavaisoflavone (7-hydroxy-3-[4-hydroxy-3-(3-methylbut-2-enyl) phenyl] chromen-4-one)

Neobavaisoflavone (7-hydroxy-3-[4-hydroxy-3-(3-methylbut-2-enyl) phenyl] chromen-4-one) (Fig. 22), is a flavonoid compound isolated mainly from *Psoraleacorylifolia* (*Leguminosae*) and *Erythrina* species. *Psoralea corylifolia* has traditionally been used in India, China and Southeastern Asian countries for the treatment of several diseases such as nephritis, asthma, cough, osteoporosis, hypertension and cardiovascular diseases [118, 119].

*Psoralea corylifolia* contains, other than neobavaisoflavone, several active compounds including psoralen, isopsoralen, psoralidin, isobavachalcone, bavachin and bakuchiol [220]. Moreover, *Psoralea corylifolia* seeds have been reported to possess a high activity against the SARS‐CoV papain‐like protease (PLpro) which is the main enzyme involved in SARS virus replication [221]. In fact, six phenolic compounds with an antiviral activity were isolated from the ethanolic extracts of *Psoralea corylifolia* identified as, bavachinin, neobavaisoflavone, isobavachalcone, 4′-O-methylbavachalcone, psoralidin and corylifol A. Isobavachalcone and psoralidin were the greatest antiviral inhibitors of SARS-CoV PLpro [222]. Actually, Neobavaisoflavone has numerous biological properties such as antibacterial, anti-fungal, anti-inflammatory, antioxidative, anti-cancer, anti-osteoporosis and anti-platelet aggregation [223, 224]. In vitro, neobavaisoflavone significantly inhibited the production of ROS, reactive nitrogen species (RNS) and cytokines: IL-1β, IL-6, IL-12p40, IL-12p70, and TNF-α in LPS+IFN-γ- or PMA-stimulated RAW264.7 macrophages [223]. Actually, *Psoralea corylifolia*, which contains neobavaisoflavone and other phenolic and non-phenolic compounds maybe used as a target for a more focused research against COVID-19 and related symptoms, since its extract was found to inhibit a human coronavirus strain [224].

## Conclusion

Natural compounds such as polyphenols with their countless properties seem to be a simple, safe and economical approach due to their numerous beneficial effects on human chronic diseases as well as on several bacterial and viral infectious diseases through their favorable effects on several cellular and molecular pathways, which leads to an enhanced body immunity against infection. This review summarized the most studied polyphenolic compounds, their main natural origin, their general role in human health focusing particularly on their role as an antiviral agent against respiratory tract infections and their perceptive role in the fight against COVID-19, related symptoms and other emergence diseases. Better design of experimental as well as human clinical studies addressing dosage and combinations of polyphenol compounds, their derivatives and other synthetized molecules are required to substantiate the benefits of such wonderful natural compounds for therapeutic and preventive purposes against infections.
